# Neurophysiological assessment of cortical activity in DEPDC5- and NPRL3-related epileptic mTORopathies

**DOI:** 10.1186/s13023-022-02600-6

**Published:** 2023-01-14

**Authors:** Madora Mabika, Kristian Agbogba, Samantha Côté, Sarah Lippé, Émilie Riou, Cécile Cieuta, Jean-François Lepage

**Affiliations:** 1 Department of Pediatrics, Faculty of Medicine and Health Sciences, Sherbrooke University, 3001-12th Avenue North, Sherbrooke, QC J1H 5N4 Canada; 2 Sherbrooke University Hospital Research Center, Sherbrooke, Canada; 3 Neuroscience of Early Development (NED), Montreal, QC Canada; 4 Research Center of the CHU Sainte-Justine Mother and Child University Hospital Center, Montreal, QC Canada; 5 Department of Psychology, Université de Montréal, Montreal, QC Canada; 6 Centre de Recherche en Neuropsychologie et Cognition (CERNEC), Montreal, QC Canada

**Keywords:** mTORC1, GATOR1, DEPDC5, NPRL3, Epilepsy, GABA, Inhibition TMS, MRS, EEG

## Abstract

**Background:**

Mutations in the GATOR1 complex genes, DEPDC5 and NPRL3, play a major role in the development of lesional and non-lesional focal epilepsy through increased mTORC1 signalling. We aimed to assess the effects of mTORC1 hyperactivation on GABAergic inhibitory circuits, in 3 and 5 individuals carrying DEPDC5 and NPRL3 mutations respectively using a multimodal approach including transcranial magnetic stimulation (TMS), magnetic resonance spectroscopy (MRS), and electroencephalography (EEG).

**Results:**

Inhibitory functions probed by TMS and MRS showed no effect of mutations on cortical GABAergic receptor-mediated inhibition and GABA concentration, in both cortical and subcortical regions. However, stronger EEG theta oscillations and stronger and more synchronous gamma oscillations were observed in DEPDC5 and NPRL3 mutations carriers.

**Conclusions:**

These results suggest that DEPDC5 and NPRL3-related epileptic mTORopathies may not directly modulate GABAergic functions but are nonetheless characterized by a stronger neural entrainment that may be reflective of a cortical hyperexcitability mediated by increased mTORC1 signaling.

**Supplementary Information:**

The online version contains supplementary material available at 10.1186/s13023-022-02600-6.

## Introduction

The mammalian/mechanistic target of rapamycin (mTOR) has been shown to play a crucial role in the developing brain, supporting the differentiation and migration of neural progenitors [1, 2], as well as myelination through the differentiation of oligodendrocytes [3]. Acumulating data converge towards the role of multiprotein complexes mTOR1 (mTORC1) activation in synaptic plasticity and memory consolidation [4–7]. Hence, the balanced activity of mTORC1 signaling is critical to the structural and functional development of the central nervous system and depends on a proper upstream regulation of the complex. Particularly, the amino acid-responsive nature of mTORC1 constitutively promotes its activation [8]; therefore, the GAP-activity-toward-RAGs complex 1 (GATOR1) acts as a strict regulator to mTORC1, inhibiting its activity. Unsurprisingly, upstream dysregulations of mTORC1 signaling have emerged as important mechanisms underlying various neurological disorders [9, 10], many of which are marked by intractable epilepsy [11, 12]. In fact, loss-of-function mutations in GATOR1 genes such as DEPDC5, NPRL2, and NPRL3) have been frequently associated with malformations of cortical development leading to focal, drug-resistant epilepsy with fair risks of sudden unexpected death in epilepsy (SUDEP) [13–18]. Consistent with what has been observed in resected human brain tissues, following epileptic surgeries, animal models of GATOR1 genes knockdown have exhibited altered neuronal networks’ morphology and excitability [19].

Rodent models have pointed DEPDC5 loss-of-function mutations as a major cause of focal epilepsy mediated by mTORC1 hyperactivation [20–22]. Such models have uncovered the role of DEPDC5 in the architectural and functional development of excitatory neuron networks. Frequently associated focal cortical dysplasia, the broad spectrum of DEPDC5-related epilepsies appears ever more relevant to the hypothesis of a ‘two-hit’ germline and somatic mutational mechanism [23–25]. More recently, a mouse model of NPRL2 and NPRL3-related mTORopathies could be assimilated to DEPDC5 knockdown models, displaying increased spontaneous seizures and cellular dysmorphism, specific to excitatory networks and astrocytes; a phenotype that is significantly alleviated with rapamycin treatment [26]. A recent study further showed a dose-dependent effect of DEPDC5 knockdown in increasing the intrinsic activity of excitatory neuron networks, mediated by mTORC1 hyperactivation [27],while the inactivation of DEPDC5 highlighted a novel role of mTORC1 upregulation in the pathogenesis of epilepsy through defects in the development of GABAergic neural networks in the zebrafish. Particularly, the inactivation of DEPDC5 downregulated GABAergic branching related genes thereby reducing seizures threshold [28]. Altogether, these data support a causative link between aberrant mTORC1 activity and cortical hyperexcitability leading to epileptic phenotypes in animal models of mTORopathies. Although animal models’ data coincide on the effects of mTORC1 hyperactivation on cortical excitability, whether the imbalance underlying epileptogenesis is mostly due to excessive excitation or lack of inhibition remains elusive.

Alterations in GABAergic function have regularly been associated with in archetypal models of epileptic encephalopathies [29–31]. Using transcranial magnetic stimulation Stern and colleagues showed a significant lack of GABAa-mediated inhibition in patients with Dravet syndrome (SCN1A mutation carriers) [32]. Alternatively, Sanchez-Carpintero and collaborators used electroencephalography (EEG) to show reduced brain gamma oscillations, putatively reflective of GABAergic interneurons activity [33], in response to auditory stimulation in Dravet children compared to healthy controls [34]. Consistent with the effects of the SCN1A gene mutation observed in mouse models of Dravet syndrome, this finding confirms impaired gamma oscillatory activity as a relevant marker of the disease. In type 1 neurofibromatosis (NF1), proton magnetic resonance spectroscopy was used to elucidate the effect of NF1 mutation on the GABAergic function and revealed a significant decrease in cortical GABA concentrations in NF1 patients compared to healthy controls; thus, clarifying the mediating role of GABAergic dysfunction on cognitive impairments observed in NF1 patients [35].

In the present study, we address markers of cortical excitability and GABAergic function in 8 DEPDC5 and NPRL3 mutations carriers, as well as in healthy controls, using transcranial magnetic stimulation (TMS), magnetic resonance spectroscopy (MRS) and electroencephalography (EEG) as complementary measures of GABAergic function. We hypothesized that the dynamic between cortical excitation and inhibition should be significantly imbalanced at the expense of inhibitory functions, in DEPDC5 and NPRL3 mutations carriers compared to healthy subjects. Particularly, we suspected this imbalanced to either origin from a dysfunction in GABAergic receptors-mediated inhibition, lower GABA concentrations, cortical hyperexcitability, or from a combination of these mechanisms.

## Material and methods

### Participants and study design

Twenty-nine (29) participants (12–65 years old; Mean: 28.9; SD: 10.9) took part in the experiment. Participants were divided into three groups: (1) 21 healthy adults subjects; (2) 3 patients with DEPDC5 mutation; (3) 5 patients with NPRL3 mutation (Table 1 for details of DEPDC5 and NPRL3 patients). Healthy adult subjects were recruited via word of mouth and public ads. Patients with DEPDC5 and NPRL3 mutations were recruited by treating neurologists from the Centre Hospitalier Universitaire de Sherbrooke (CHUS). All participants were screened for TMS and MRI contraindication using standard questionnaires [36] prior to testing. The presence of metallic items or device in the body and any history of traumatic brain injury were considered as absolute exclusion criteria. Signed informed consent was obtained from all adult participants and from minor participants’ legal representatives. The study was approved by the local ethics committee (Comité d’Éthique de la Recherche du CIUSSS de l’Estrie – CHUS) and met the standards of the 1964 Helsinki Declaration. Participants were invited to the CHUS research center for a single visit. In a random order, each participant was submitted to a 1 h transcranial magnetic stimulation (TMS) session, a 1 h magnetic resonance spectroscopy imaging and 1h30 EEG recording. When testing epileptic patients, foam mats were installed in the TMS room, and pediatric neurologists were available on call (CC, ER) for immediate support in the unlikely event of a seizure during the experiment, either spontaneous or related to the use of TMS.

**Table 1:** Characteristics of DEPDC5 and NPRL3 participants

ID	Sex	Age (years)	Medication (mg/day)
DEPDC5_01	F	33	ESL (400), CLB (10), LTG (150)
DEPDC5_02	F	48	None
DEPDC5_03	F	68	None
NPRL3_01	F	39	None
NPRL3_02	M	12	None
NPRL3_03	F	14	CBZ (400)
NPRL3_04	F	16	CBZ (1200); CLB (1)
NPRL3_05	F	36	None

*CBZ* Carbamazepine, *CLB* Clobazam, *ESL* Eslicarbazepine acetate, *LTG* Lamotrigine

### Transcranial magnetic stimulation (TMS)

#### Experiment

TMS was performed using a Magstim Bistim2 apparatus (The Magstim® BiStim^2^, Wales, UK) equipped with a 70 mm figure-of-eight coil. We were inspired by the work of Morin-parent and collaborators in fragile X syndrome to asses intracortical activity following the same TMS paradigms and using similar experimental settings [37]. A test stimulus (TS), set at 125% of the resting motor threshold (rMT—established using the relative frequency criterion [38]) was applied in single-pulse procedures assessing cortical excitability (100–150% of rMT pulses in 10% steps) and corticospinal silent period (during voluntary muscle contraction at 20% of maximum force). TS was paired with a conditioned stimulus (CS), set at 70% of rMT, with different interstimulus intervals (ISI) in procedures assessing: short intracortical inhibition (SICI—2 ms, 4 ms ISI), intracortical facilitation (ICF—10 ms, 15 ms ISI), Short intracortical facilitation (SICF—3 ms ISI) and Long intracortical inhibition and facilitation (LICI/F—100 ms and 220 ms ISI). Fifteen trials were obtained for each of these measures, providing a Chronbach’s alpha > 0.90 [39].

#### Data processing

**Fig. 1:**
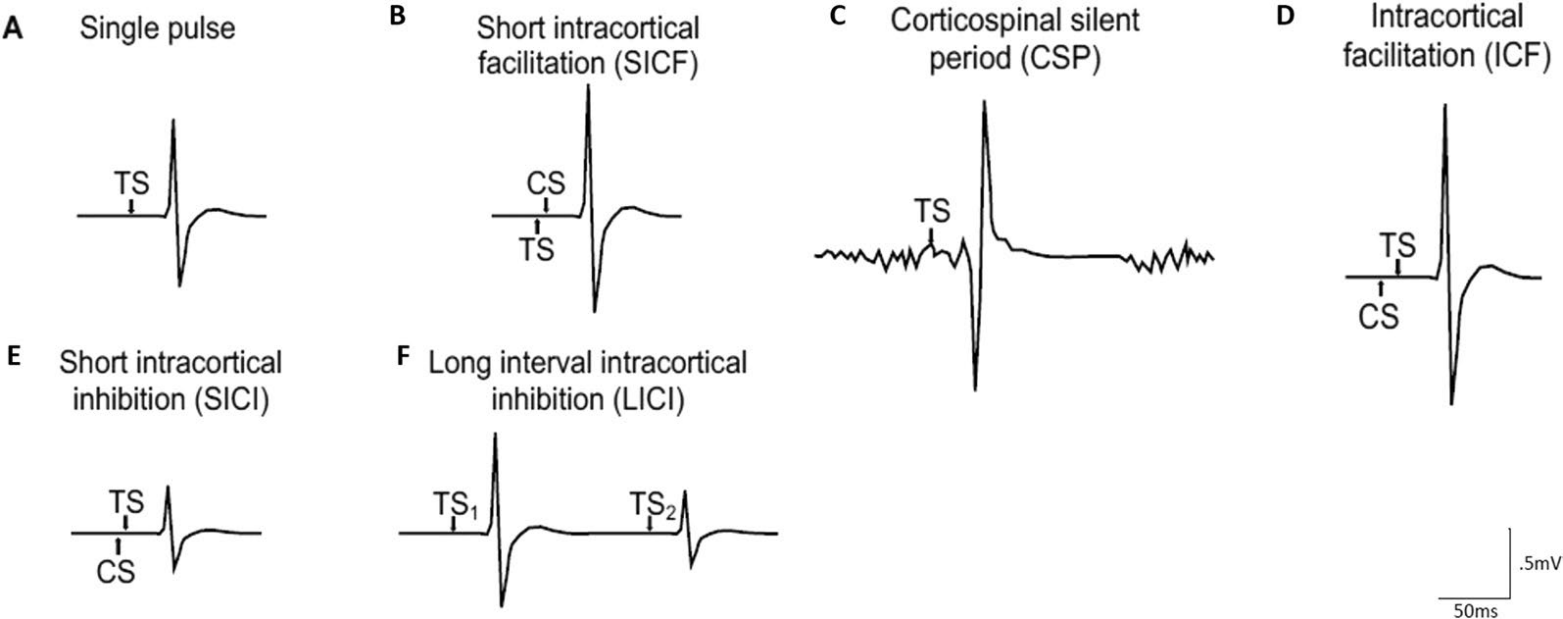
Standard plot of motor evoked potential as measured by electromyography for different transcranial magnetic stimulation protocols. *CS* conditioned stimulus, *TS* test stimulus

TMS data was processed offline using LabChart 8 (ADInstruments) on a Macintosh Powerbook. TMS-elicited peak-to-peak MEP amplitudes were measured for all protocols (Fig. 1), except for the CSP. Average peak-to-peak MEP amplitudes were calculated for baseline MEP, SICI (2–4 ms), ICF (10–15 ms), and SICF (3 ms), For SICI, ICF and SICF, ratios were computed over baseline MEP to obtain indices of inhibition (< 1) or facilitation (> 1). For each trial of the LICI (100-220 ms), the amplitude of the second MEP was divided by the amplitude of first to compute ratios of inhibition, which were then averaged. Average peak-to-peak MEP amplitudes were also calculated for each intensity of the cortical excitability gradient to produce input/output curves. The CSP was established as the average duration in ms between TMS pulse and the return to baseline EMG activity.

### Magnetic resonance spectroscopy (MRS)

#### Experiment

**Fig. 2:**
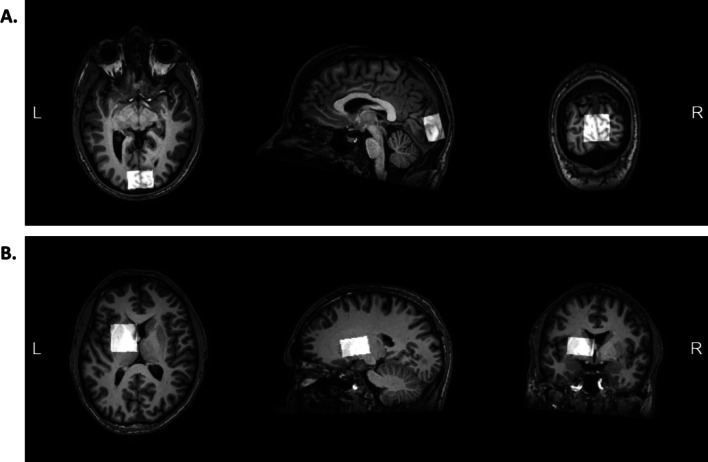
Anatomical landmarks for single voxel spectroscopy (T1-weighted images). **a** The first voxel was located bilaterally at the primary visual cortex. **b** The second voxel was located at the globus pallidus, in the left hemisphere

Magnetic resonance imaging took place at the molecular imaging center of Sherbrooke (CIMS), located at the CRCHUS. MRI and MRS data were acquired using a whole-body 32-channel head coil scanner (Ingenia 3.0 T MR system, Philips, USA). Each session began with the acquisition of anatomical images of the whole brain in T1-weighted contrast. These images were then used as a benchmark for the placement of the spectroscopic voxels of interest (VOI), in respect with the following parameters: VOI size = 20*30*30 mm3; VOI orientation: transverse. Two single voxel spectroscopy measurements were obtained, the first VOI was placed in the occipital cortex bilaterally, and the second VOI was placed on the basal ganglia’s globus pallidus of the left hemisphere (Fig. 2). The MEscher-Garwood Point RESolved Spectroscopy (MEGA-PRESS) sequence [40] was used to measure the signals of the metabolites of interest, applying the following parameters: TR (repetition time) = 2000 ms; TE (echo time) = 68 ms; Excite flip angle = 90°; Refocus flip angle = 180°. Water suppression was first performed applying a water-suppressing band at 4.7 ppm, using the excitation technique.

#### Data processing

Data were processed with the Gannet3.1 (GABA-MRS Analysis Tool) pipeline in MATLAB. Phased-array channel combination and phase cycling averaging were automatically performed during data exportation. Tissue segmentation was performed using SPM to acquire grey matter, white matter, and cerebrospinal contents within each voxel. GABA and glutamate concentrations were corrected for voxels' gray and white matter contents and normalized to groups-average contents for all the participants [41] (Additional file 1: Figure 1).

### Electroencephalography (EEG)

#### Experiment

The EEG recording was performed using a 64 channel ActiCap Slim (Brain Products©). Electrode wires were gathered in two sets of 32 bundles through splitter boxes then routed to two standard BrainAmp amplifiers (Brain Vision Solutions©). The signal was digitized through a USB 2 adapter connected to the amplifiers then recorded at the computer using Brain vision recorder at a 1000 Hz sampling rate. Participants were comfortably seated on a chair with their back to the EEG installation. Auditory stimuli were presented using Psyscope XB77 software. Sounds were transmitted through audio speakers placed bilaterally, approximately 25 cm from their ears, with sound volume set at 65. During the procedure, participants watched a silent movie to facilitate collaboration and avoid movement artefacts as much as possible.

The task was similar to the one used by Ethridge and colleagues [42, 43] where an auditory chirp stimulus made of a pure tone (1000 Hz) which amplitude was modulated by a sinusoid increasing linearly in frequency from 0 to 120 Hz over 2000 ms was presented 160 times, with ISI varying randomly between 1500 and 2500 ms.

#### Signal processing

Signal preprocessing was performed using the EEGLAB toolbox in MATLAB. A 0.5–150 Hz bandpass and a 60 Hz notch filter were applied. Prior further processing steps, 19 electrodes with poor signal quality were removed in the anterior-frontal, frontotemporal, temporal, parietal, and parieto-occipital regions, for all participants. Upon visual inspection additional noisy electrodes were manually removed, and all electrodes for which the signal amplitude had a standard deviation of less than 2 mV and greater than 120 mV were automatically removed. On average 40 (± 4) electrodes were conserved for healthy subjects and 37 (± 4) for patients. Blinks, saccades, and cardiac activity-related artifacts were isolated using Independent Component Analysis (ICA), and the artifacted components were then rejected, blind to participant groups. Data was segmented into 4.5 s epochs (1 s pre-stimulus and 3.5 s post-stimulus). Artifact rejection was performed semi-automatically: epochs containing amplitudes > 200 µV and < − 200 µV were tagged and artifacted segments were manually removed during subsequent visual inspection, accounting for all remaining artifacts (movement etc.). On average, 140 (± 19) epochs were kept for controls and 131 (± 29) epochs were kept for patients after this step. For data quality and interpretation purposes, all subsequent analyses focused on four defined regions of interest (ROI), defined by an average of a specific group of electrodes located over the frontal region (Fz: F3, F1, Fz, F2, F4), central region (Cz: C1, Cz, C2, FC1, FCz, FC2), parietal region (Pz: P3, P1, Pz, P2, P4); and occipital region (Oz: O1, Oz, O2, PO3, POz, PO4) (Additional file 1: Figure 2).

#### Time–frequency analyses

**Fig. 3:**
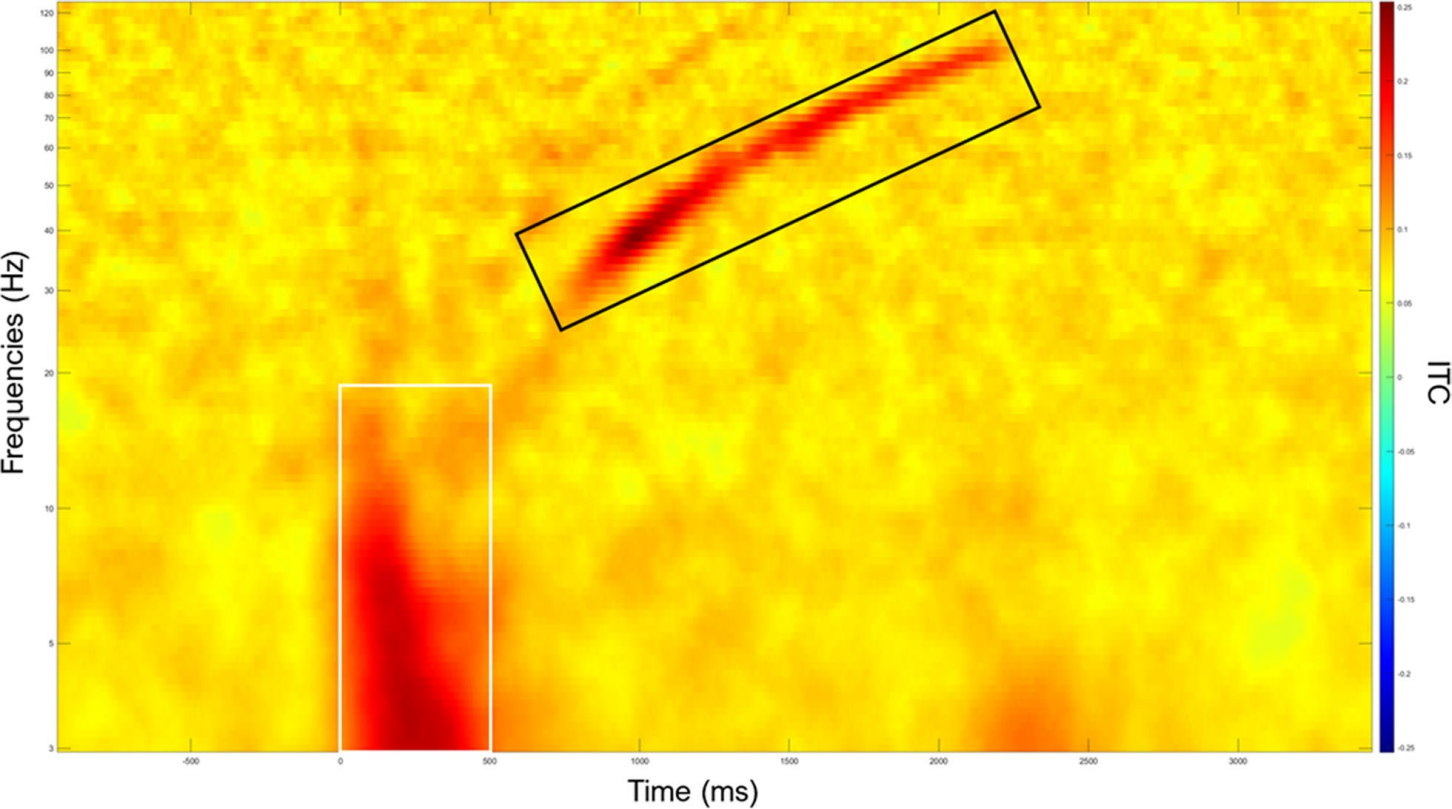
Chirp onset and entrainment responses mask regions. The present map represents the whole sample’s mean inter-trial coherence of the chirp-evoked oscillatory response, with rectangles focusing on areas of maximum inter-trial coherence. The white rectangle shows the chirp onset response region (0–500 ms), and the black rectangle shows the chirp entrainment response region (750–2500 ms), within which time–frequency points with ITC ≥ 0.13 were selected and masked

Time–frequency analyses were performed in EEGlab (implemented in MATLAB). Event-related spectral perturbation (ERSP) and inter-trial coherence (ITC) indices were used to quantify frequencies’ amplitude and phase coherence across trials. Whole sample ERSP and ITC maps were generated for all regions of interest. The complex Morlet wavelet transform was used to get the event-related spectral perturbation (ERSP) and inter-trial coherence (ITC) components of the signal. Evoked responses were marked by greater phase-locking over the frontal region, from which two masks were then generated by selecting all points with an ITC > 0.13. A first masks was generated for the response to the chirp onset and a second mask was generated for the entrainment response to the chirp sound (Fig. 3). These masks fitted into fixed time ranges (onset response: 0–500 ms; entrainment response: 750–2250 ms) which allowed us to extract within-masks frequencies’ amplitude and synchronization indices from all ITC maps across subjects. For all regions of interest, these values were then modeled as curves indicating the average ERSP increase (logarithmic gain in decibels) on one hand; and the average ITC increase (synchronization gain) on the other hand, in all subjects.

### Statistical analyses

Analyses were performed using the Jamovi statistical software (The Jamovi project (2021), version 2.2.1) and in MATLAB. For all TMS, EEG and MRS measurements, assumption of normality and equality of variances were checked using the Shapiro–Wilk and Levene tests respectively. Differences between groups were computed using One-way ANOVA. For both masks, group-average ERSP and ITC were calculated across trials, for the 4 regions of interest. For the chirp onset response mask, areas of maximum power (ERSP) and phase-locking (ITC) were identified (based on the curves of average amplitude and synchronization increases) and set for analyses for frequencies ranging from: 3 to 8 Hz (Theta band), 8 to 14 Hz (Alpha band), and 14 to 17 Hz (Beta band). For the chirp entrainment response, areas of maximum power (ERSP) and phase-locking (ITC) were similarly identified and set for analyses for frequencies ranging from: 30 to 55 Hz (low gamma) and 65 to 100 Hz (high gamma). Differences between groups were then computed in Jamovi using One-way ANOVA and were considered significant for *p* < 0.05. Post hoc analyses were conducted with Tuckey and Games-Howell tests.

## Results

The TMS session could not be completed in one participant with DEPDC5 mutation, and one participant with NPRL3 mutation was excluded based on medical advice from the treating neurologist. One NPRL3 carrier refused to proceed to the MRS experiment. Due to technical issues, EEG data acquisition could not be completed for two NPRL3 carriers.

### TMS

**Fig. 4:**
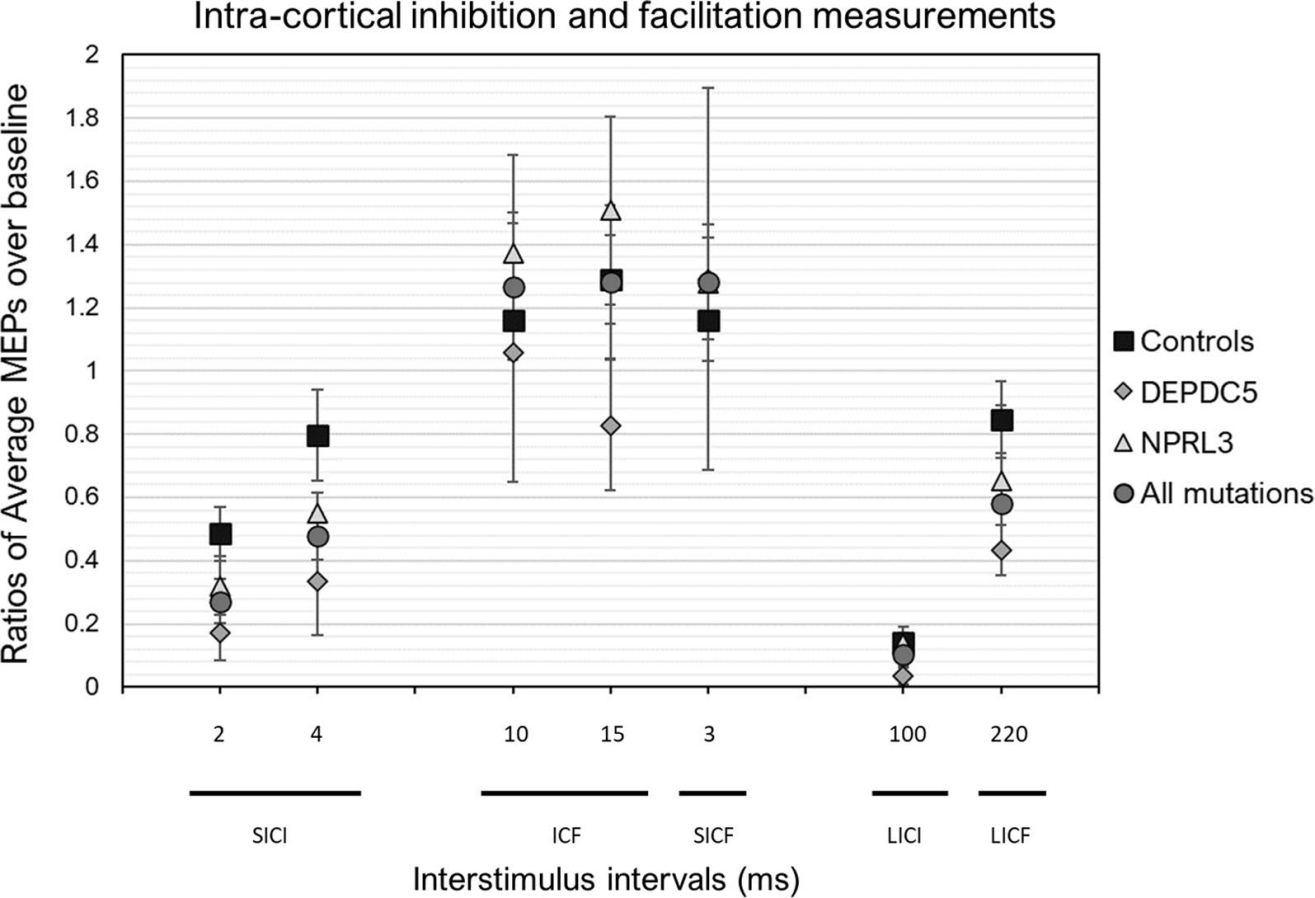
Quantification of intra-cortical inhibition and facilitation. Ratio values are indicated as mean ± standard error. SICI = Short interval cortical inhibition. ICF = Intra-cortical facilitation. SICF = Short Interval Cortical Facilitation. LICI = Long Interval Cortical Inhibition. LICF = Long Interval Cortical Facilitation. MEP = Motor Evoked Potentials

For all pair-pulsed TMS measures, the one-way ANOVAs showed no significant difference between the three groups (all p > 0.05), suggesting no sizable changes in intracortical excitation and inhibitory mechanisms (Fig. 4). Similarly, no difference was observed for TMS measures pertaining to cortical excitability and cortical silent period (Additional file 1: Figures 3 & 4).

### MRS

**Fig. 5:**
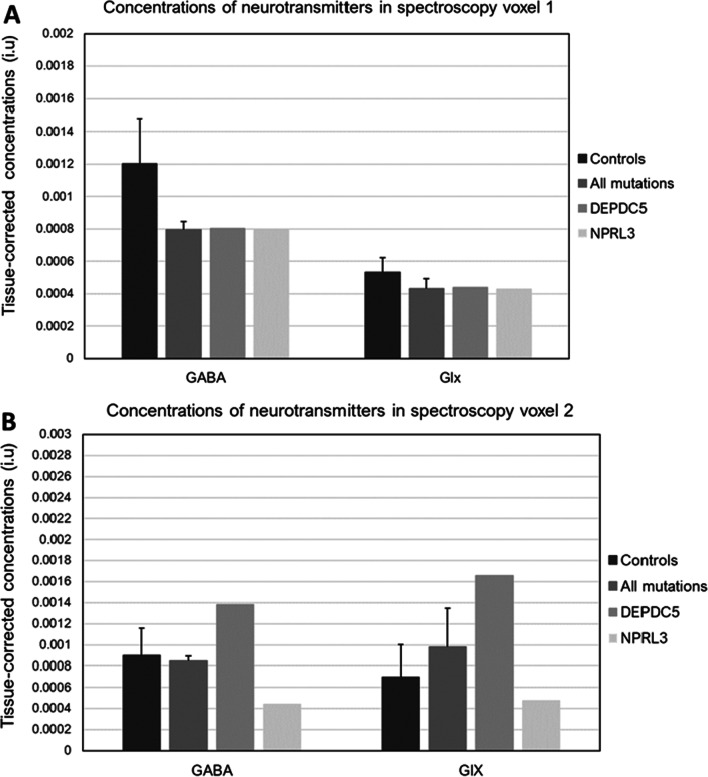
Neurotransmitters’ concentrations. Concentrations in the primary visual cortex (**a**) and left globus pallidus (**b**). Concentrations are indicated as mean ± standard error

The three groups did not differ in terms of tissue content proportions within both spectroscopy voxel (*p* > 0.05). No significant differences were observed with regards to extracellular GABA or Glx concentrations in either voxel (Fig. 5).

### EEG

Significant differences in EEG onset and entrainment responses to the chirp sound were found in DEPDC5 and NPRL3 individuals as compared to healthy subjects, particularly in the theta (ERSP: F = 6.518, p = 0.006; ITC: F = 6.482, p = 0.006) and gamma (ERSP: F = 10.1, p < 0.001; ITC: F = 6.42, p = 0.006) frequency ranges (Additional file 1: Figures 5–7). However, we must keep in mind that each mutation seems to affect carriers' GABAergic functions differently as some display epileptic phenotypes whereas other are seizure-free (Table 1). In addition to the lack of statistical power, one may question the relevance and validity of such differences.

In this context, using a descriptive, rather than a statistical approach, individual EEG results are presented here, comparing the results of one epileptic patient and one seizure-free carrier in each of the DEPDC5 and NPRL3 subgroups. To our knowledge, none of the DEPDC5 and NPRL3 carrier and epileptic patients had a history of cortical dysplasia. In the absence of further clinical data (EEG recording, MRI data) that would have directed the analysis of our results, we focus here on the regions that were prone to the most significant differences within theta and low gamma frequency ranges, during group-average comparisons, namely the frontal and parietal regions.

**Fig. 6:**
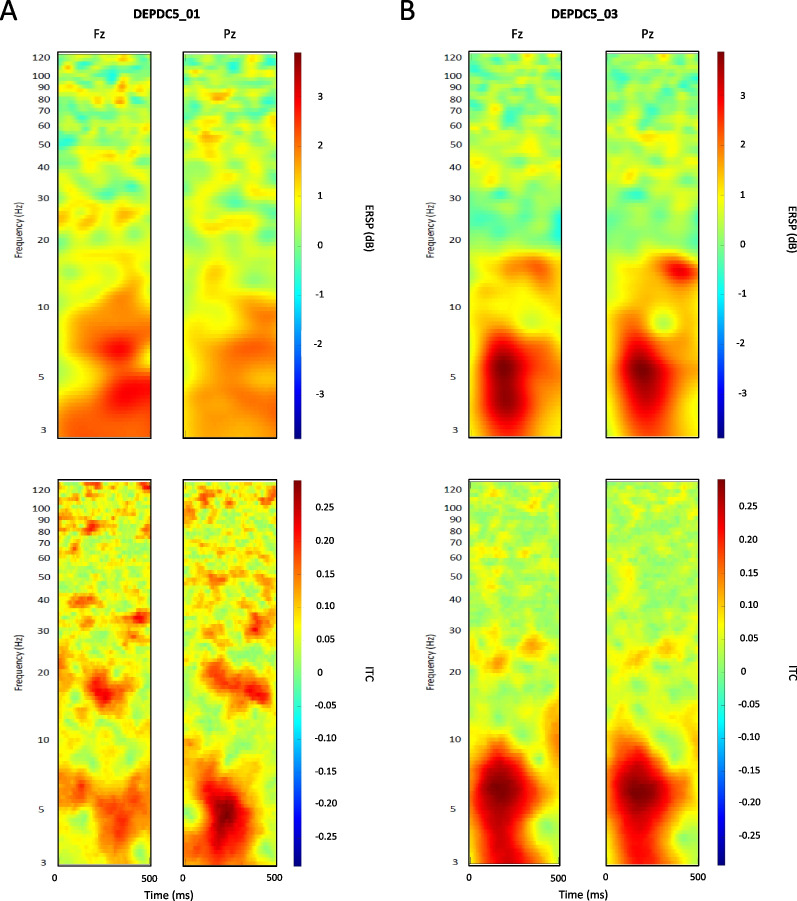
Oscillatory responses to chirp sound onset in DEPC5 epileptic patient vs. seizure-free carrier. Mean ERSP and ITC map sections show oscillatory responses to stimulus onset across trials in one epileptic patient (**a**) and one seizure-free carrier (**b**) Variations in frequencies’ spectral power and phase coherence are indicated by the color bars

When comparing DEPDC5 epileptic patient and seizure-free carrier, oscillatory responses modulated by the stimulus onset could be observed within low frequency bands, in both individuals. Over the frontal and parietal regions, these responses were marked by increased spectral power and phase coherence that appear more significant in the seizure-free carrier compared to the epileptic patient, as indicated by the color bar (Fig. 6). On one hand, In the epileptic patient, the stimulus onset induced oscillatory responses associated with 2 dB < ERSP < 3 dB in the frontal region and 2 dB < ERSP < 3 dB in the parietal region, within 3–10 Hz (Fig. 6a). On the other hand, both frontal and parietal oscillatory responses are associated with ERSP ≥ 3 dB in the seizure-free carrier, particularly within theta (3–8 Hz) frequency range (Fig. 6b). In response to the stimulus onset, the seizure-free carrier also showed a significantly more increased theta phase coherence (ITC ≥ 0.25) over the frontal and parietal regions (Fig. 6b), which was only observed over the parietal region in the epileptic patient (Fig. 6a).

**Fig. 7:**
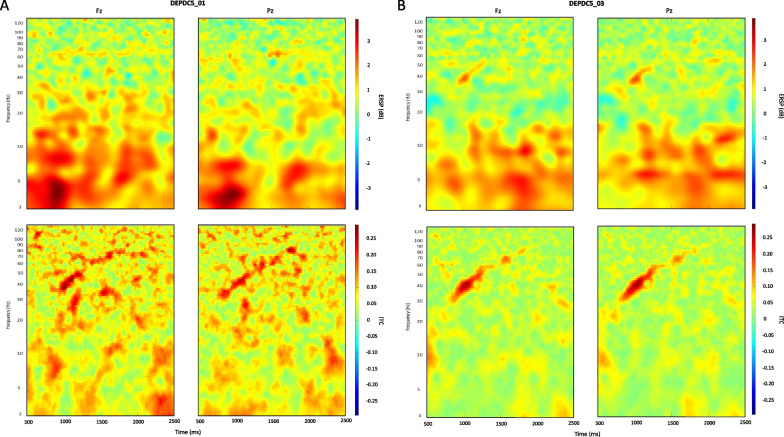
Oscillatory entrainment responses to chirp sound in DEPC5 epileptic patient versus seizure-free carrier. Mean ERSP and ITC map sections show entrainment responses to the stimulus across trials in one epileptic patient (**a**) and one seizure-free carrier (**b**). Variations in frequencies’ spectral power and phase coherence are indicated by the color bars

Similar patterns are observed for entrainment responses to the chirp sound across trials, pertaining to high frequency oscillations. In the seizure-free DEPDC5 carrier, mean ERSP and ITC map sections highlight a distinct spectral power increase within 35–50 Hz, and a greater phase coherence increase within 30 to 60 Hz, with maximum ITC values around 40 Hz gamma band (Fig. 7b). Like the seizure-free carrier, the mean ITC map highlights a significant increase phase-coherence, with maximum ITC values around 40 Hz gamma band, in the epileptic patient (Fig. 7a). However, entrainment responses to the chirp sound across trials caused no remarkable increase in gamma spectral power in the epileptic patient (as suggested by the absence of this characteristic diagonal shape).

**Fig. 8:**
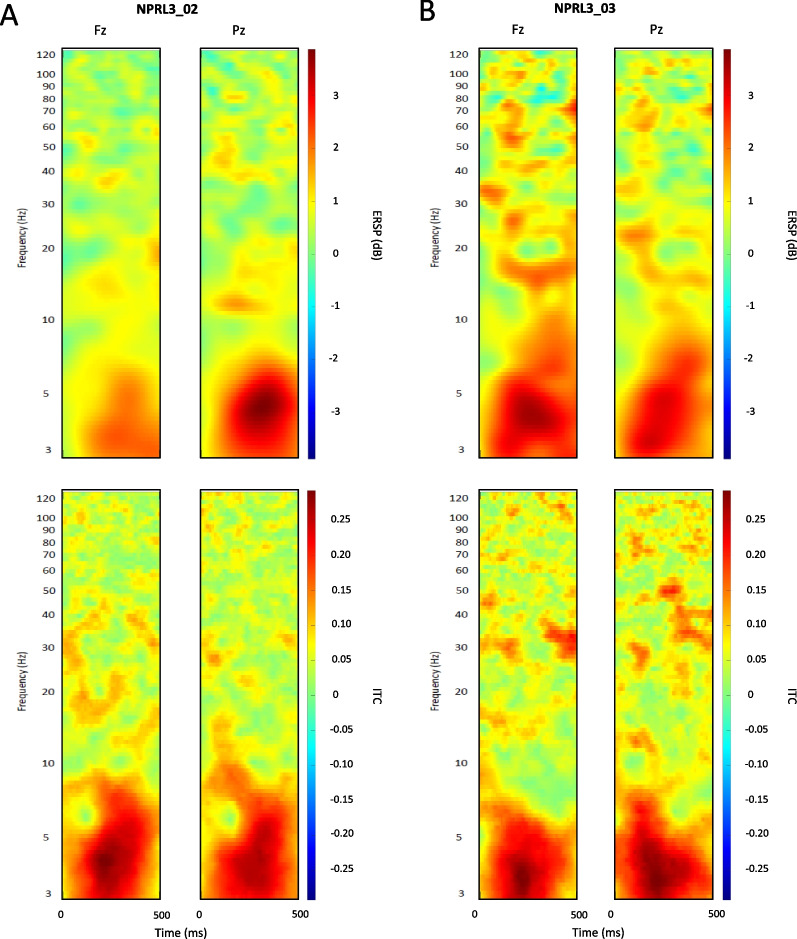
Oscillatory responses to chirp sound onset in NPRL3 epileptic patient vs. seizure-free carrier. Mean ERSP and ITC map sections show oscillatory responses to the stimulus onset across trials in one seizure-free carrier (**a**) and one epileptic patient (**b**) Variations in frequencies’ spectral power and phase coherence are indicated by the color bars

In the case of NPRL3 mutation, EEG responses to the chirp sound appear quite similar to that described in DEPDC5 mutation. The stimulus onset caused a significant increase in theta spectral power, with maximum ERSP values (≥ 3 dB) over the parietal region in the unaffected carrier (Fig. 8a) and over the frontal region in the epileptic patient (Fig. 8b). Theta phase-coherence was equally subject to a maximum increase (ITC ≥ 0.25) in both epileptic and unaffected NPRL3 carrier (Fig. 8a, b), over the frontal and parietal regions.

**Fig. 9:**
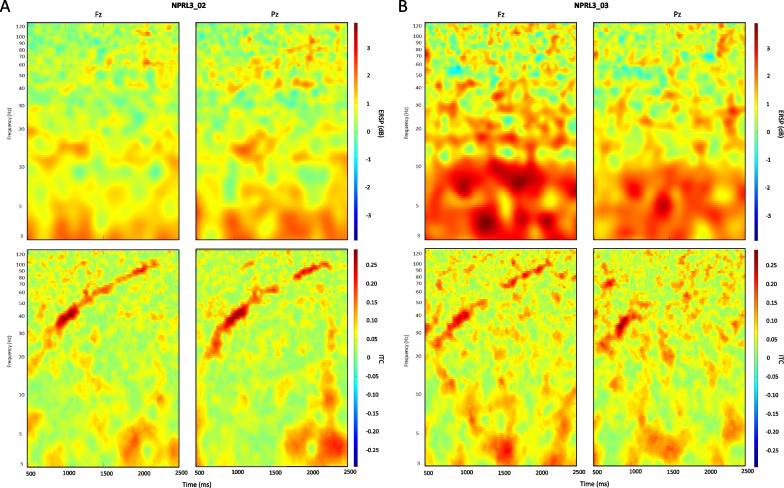
Oscillatory entrainment responses to chirp sound in DEPC5 epileptic patient versus seizure-free carrier. Mean ERSP and ITC map sections show entrainment responses to the stimulus across trials in one seizure-free carrier (**a**) and one epileptic patient (**b**). Variations in frequencies’ spectral power and phase coherence are indicated by the color bars

As in DEPDC5 individuals, Fig. 9 shows that entrainment responses to chirp sound are pertaining to the gamma frequency band in NPRL3. Particularly, mean ITC maps show distinct chirp-modulated phase coherence increase within 30 to 100 Hz. In the frontal and parietal regions, both NPRL3 epileptic patient and unaffected carrier display aberrant gamma phase coherence, as depicted by the characteristics diagonal shape, with maximum ITC (≥ 0.25) values reached between 35 and 45 Hz.

Interestingly, we observed in both mutations that entrainment responses to the chirp sound across trials are marked by a significant increase in theta spectral power (ERSP ≥ 3) specific to the epileptic individual (Fig. 7a) as compared to the unaffected carrier (Fig. 9b).

## Discussion

Genes coding for the mTORC1-regulating complex GATOR1 proteins are strongly associated with various phenotypes across the spectrum of inherited focal epilepsies, that may also involve malformations of cortical development [44]. So far, animal models and resected human brain tissue analyses have provided groundwork for confirming the mediating role of mTORC1 hyperactivation in epileptogenesis, essentially following DEPDC5, but also NPRL2 and NPRL3 loss-of-function mutations [19, 45, 46]. However, the exact mechanisms by which this increased mTORC1 activity disrupts the balance between cortical excitation and inhibition in clinically observed phenotypes remain to be established. In the present study, we aimed to assess whether mTORC1 hyperactivity following DEPDC5 and NPRL3 mutations involves changes in the GABAergic system, the primary inhibitory neurotransmitter of the central nervous system. We used a combination of non-invasive techniques to probe three distinct elements pertaining to GABAergic function.

First, we found that TMS measures of intracortical inhibition were not different in DEPDC5 and NPRL3 carriers, compared to healthy controls. Interpretation of unchanged GABAergic receptor-mediated cortical inhibition here should consider in insight from other TMS studies in neurogenetic conditions marked by refractory epilepsy. In that regard, these findings differ from what is observed in patients with Dravet syndrome [32] and fragile X syndrome (FXS) [37], two disorders associated with epilepsy, where reduced GABAa-mediated inhibition was observed. Overall, our data do not support the hypothesis of a mutation effect on GABAr-mediated inhibition. Despite similarities in the clinical phenotypes, discrepancies between data relating to the GABAergic system in the fragile X syndrome and our results can be explained by the different genetic mechanisms involved in the etiology of these syndromes. However, we must keep in mind that the small sample size remains the greatest limitation here and likely explains the lack of significant results.

To our knowledge, no other study has addressed the neurochemical effects of GATOR1 genes mutations in the development of epileptic phenotypes so far, in animal or in human. In the present study, 1H-MRS revealed no significant group differences in GABA concentrations. To test whether the epileptic phenotypes were attributable to an excess of excitatory neurotransmitters, we similarly measured glutamate and glutamine concentrations. Here again, the absence of significant group difference suggests no effect of GATOR1 gene mutations on cortical and subcortical neurochemical balance. However, some limitations are to be considered with these results; first and foremost, the approach used here does not differentiate intracellular from extracellular GABA concentrations [47]. To continue, although most carriers were seizure-free, few of our participants were treated with combination of AEDs that are known to act on the GABAergic system, hence affecting cortical and subcortical GABA levels, directly or indirectly. A mutation effect on cortical and subcortical GABA concentrations might have been observed, had our study benefited from a larger sample. So far, MRS has not been extensively used to further assess neurochemical changes in focal, inherited epilepsies, and the scarce available data appear inconsistent in temporal lobe epilepsy and focal cortical dysplasia [48, 49]. Although GATOR1 genes, particularly DEPDC5, have been associated to mesial and lateral temporal lobe epilepsy and more frequently to FCD-associated epilepsy, no information about the genetic profile of the TLE and FCD patients is provided in these studies. Their conclusions must therefore be considered with caution.

The sheer nature of epileptic seizures, which are characterized by a large-scale hypersynchronous activity of neuron populations [50], make the use electroencephalography (EEG) particularly relevant in the study of focal epilepsies, such as mTOR-related epileptic syndromes. The auditory task used in this study meets the requirements for recording EEG oscillations that fall in the frequency range of theta and gamma rhythms. The 1–120 Hz-modulated Chirp sound elicits EEG responses generated in this same frequencies’ interval, with no active participation required from the subjects. In the analysis, we used indices that simultaneously addressed the magnitude of and phase of the decomposed EEG spectrum. For each epoch, the event-related spectral perturbation (ERSP) measures the mean dynamic changes in frequencies’ amplitude (in dB) over time, while the inter-trial coherence (ITC) reflects the consistency of the cerebral response across trials, showing the degree of frequencies’ phase alignment in response to the stimulus [51, 52].

Theta oscillations represent one of the most regular EEG activities that can be recorded, emerging both from cortical and subcortical regions. Due to the high density of its neural layers, the hippocampus is considered one of the main sources of EEG theta. In the cases presented here we describe EEG theta features common to both mutations and specific to epileptic patients as compared to unaffected carriers. Although it is worth mentioning that amplitude generally increase as frequencies decreases, here, the presence of aberrant theta spectral power in both DEPDC5 and NPRL3 epileptic patients align with the growing body of research highlighting aberrant theta oscillations; in adults with genetic and neurological disorders [53] including FXS [43, 54] and generalized epilepsy [55, 56]. Considering the GATOR1 genes mutations effect on the mTOR signaling pathway, one can suspect the mTORC1 hyperactivation to impede the cognitive and memory functions that rely on theta; in the present case, the processing of auditory information. However, it is worth considering the potential effects of medications regarding these results. Indeed, neuropharmacological studies of AEDs have proved compounds such as carbamazepine to cause a power increase of low frequency oscillations, particularly theta oscillations, not only in healthy individuals but also in epileptic patients [57, 58]. This may explain the significant increase in theta power induced by the stimulus onset, particularly in the case of the NPRL3 patient presented here, treated with carbamazepine.

While pyramidal cells primarily contribute to the generation of hippocampal theta oscillations, it has also been observed that interneurons, particularly GABAergic interneurons, act as essential modulators of theta oscillations [59–61], regulating the firing tone of pyramidal cells and promoting local network synchronization [62–64], particularly during seizures [65]. It is now-established that GABAergic interneurons act as the main generators of high-frequency oscillations [66]. Particularly, the parvalbumin-expressing GABAergic interneurons, characterized as fast-spiking, are typically associated with cortical but also hippocampal gamma oscillations through which they rhythmically inhibit networks of pyramidal cells [33, 67–69]. It is generally acknowledged that the coherence of such high-frequency oscillations supports a dynamic communication between neural ensembles across brain regions that is essential to cognitive functions [70–72]. Unsurprisingly, changes in gamma activity are observed in intractable epilepsy [73]. Recent work by Sato and colleagues focused on the spatiotemporal characteristics of gamma oscillations in FCD-related epilepsy. Analyzing intracranial EEG data, they highlighted dynamic changes in GABAergic interneurons synchronization underlying epileptiform activity and seizure generation [74]. Contrasting individual data in each of the mutations, we observed that EEG gamma responses to the chirp sound are significantly more coherent in unaffected carriers compared to epileptic patients. In the case of DEPDC5 mutation, not only EEG gamma responses are more coherent, but they are also of greater amplitude in the unaffected carrier, suggesting a hyper-synchronization of the neural networks responsible for these oscillations, presumably due to faulty GABAergic interneurons activity. Yet, it is worth mentioning that in epileptic patients this effect may be attenuated by AEDs.

In each of the mutation, we also note that theta and gamma are aberrantly co-occurring in entrainment responses to the stimulus across trials, as evidenced by the significant increase in theta spectral power concomitant with maximum increases in gamma phase-locking. This is particularly remarkable in epileptic patients, compared to seizure-free carriers. Interestingly, recordings of single units and local field potentials in animals have showed a match between neocortical gamma power and hippocampal theta phase, further indicating the entrainment of neocortical neuron ensembles to high-frequency oscillations by low-frequency oscillations [75, 76]. In fact, alterations in theta-gamma cross-frequency coupling are now considered a biomarker of neurologic conditions that share GABAergic dysfunction, especially Alzheimer’s disease and epilepsy [77, 78]. Notwithstanding the potential effect of AEDs on theta spectral power as previously mentioned, this observation raises questions regarding a potential effect of DEPDC5 and NPRL3 mutations on the coupling of theta and gamma activities leading to a failure in the functions they support With this in mind, and accounting for the nature and main features of mTORopathies, it can be hypothesized that although mutations in the GATOR1 genes do not directly affect GABAergic functions at the synaptic level, they likely promote electrophysiological changes within neuron ensembles, including GABAergic circuits, through increased mTORC1 activity, resulting in cortical hyperexcitability driven by hippocampal-neocortical interactions [79]. Although, the mechanisms by which GATOR1 genes mutations create changes in human cortical excitability remain unclear, single-cases comparison here suggest that DEPDC5 and NPRL3 mutations effects on cortical excitability, may vary with phenotypic profile.

## Conclusion

Alterations in GABAergic function are likely common driving mechanisms in generalized and focal epileptic syndromes. However, these changes may occur at different levels depending on the etiology of the disease. Consistent with previous work and current knowledge of focal epilepsy, TMS and MRS here revealed no changes in physiological and biochemical factors of GABAergic transmission in DEPDC5 and NPRL3-related epileptic mTORopathies. In contrast, aberrant EEG theta and gamma oscillations, in response to auditory stimulation, were remarkable in DEPDC5 and NPRL3 seizure-free carriers and patients. This finding may be relevant as an electrophysiological sign of cortical hyperexcitability involved in epileptogenesis through increased mTORC1 activation.

## Supplementary Information

**Additional file 1. Supplementary figure 1.** Tissue-content normalization formula for GABA and glutamate quantification. **Supplementary figure 2.** Defined regions of interest for EEG data analyses. **Supplementary figure 3.** Curves of cortical excitability. **Supplementary figure 4.** Average cortical silent period in each group. **Supplementary figure 5.** Group-average changes in frequencies’ ITC during the Chirp onset. **Supplementary figure 6.** Group-average changes in frequencies’ ERSP in response to the chirp sound. **Supplementary figure 7.** Group-average changes in frequencies’ ITC in response to the chirp sound.

## Data Availability

The datasets generated and analysed during the current study are available from the corresponding author on reasonable request.

## Abbreviations

mTOR: Mammalian/mechanistic target of rapamycin
GATOR1: GAP-activity-toward-RAGs complex 1
DEPDC5: DEP (Dishevelled, Egl-10 and Pleckstrin)-Domain Containing 5
NPRL3: Nitrogen Permease Regulator Like 3
SUDEP: Sudden unexpected death in epilepsy
TMS: Transcranial Magnetic Stimulation
SCN1A: Sodium Channel voltage-gated type 1 alpha subunit
SICI: Short interval cortical inhibition
LICI: Long interval cortical inhibition
ICF: Intra cortical facilitation
SICF: Short interval cortical Facilitation
CSP: Cortical silent period
ASD: Autism spectrum disorder
FXS: Fragile X syndrome
NMDAr: N-methyl-D-aspartate receptors
^1^H-MRS: Proton-Magnetic Resonance Spectroscopy
GABA: Gamma-aminobutyric acid
Glx: Glutamate + glutamine
NF1: Neurofibromatosis type 1
AEDs: Anti-epileptic drugs
FCD: Focal cortical dysplasia
TLE: Temporal lobe epilepsy
ERSP: Event-related spectral perturbation
ITC: Inter-trial coherence
