# Supplementary material for: Neurophysiological assessment of cortical activity in DEPDC5- and NPRL3-related epileptic mTORopathies
Source: Orphanet J Rare Dis. 2023 Jan 14;18:11. doi: 10.1186/s13023-022-02600-6 (PMC9840333; doi:10.1186/s13023-022-02600-6)
Supplement: Supplementary file 1 — Additional file 1. Supplementary figure 1. Tissue-content normalization formula for GABA and glutamate quantification. Supplementary figure 2. Defined regions of interest for EEG data analyses. Supplementary figure 3. Curves of cortical excitability. Supplementary figure 4. Average cortical silent period in each group. Supplementary figure 5. Group-average changes in frequencies’ ITC during the Chirp onset. Supplementary figure 6. Group-average changes in frequencies’ ERSP in response to the chirp sound. Supplementary figure 7. Group-average changes in frequencies’ ITC in response to the chirp sound. [file 13023_2022_2600_MOESM1_ESM.docx]

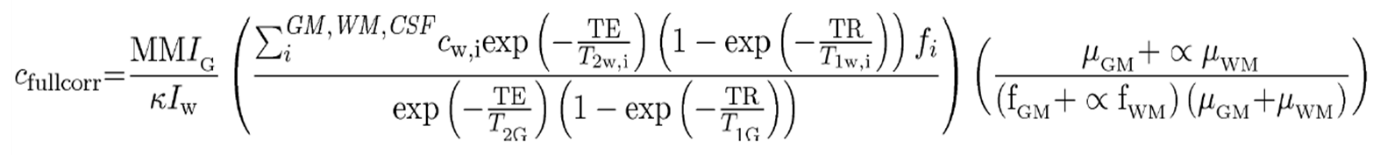


**Supplementary figure 1. Tissue-content normalization formula for GABA and glutamate quantification.** I_G_ = GABA area. I_w_ = water area. GM = grey matter. WM = white matter. CSF = cerebrospinal fluid. κ = editing efficiency of GABA (0.5). c_w,i_ = visible water concentration in each tissue type (43.3 mol/dm^3^ in GM; 36.1 mol/dm^3^ in WM; 53.8 mol/dm^3^ in CSF). MM = co-edited macromolecules signal correction factor. TE = echo time (68ms). TR = repletion time (2000ms). T_1G_, T_2G_ = T1 and T2 relaxation time constant for GABA (88ms for T1 and 800ms for T2). T_1w,i_ = T1 relaxation time constant for GM (1331ms), WM (832ms), and CSF (3817ms). T_2w,i_ = T2 relaxation time constant for GM (110ms), WM (79.2) and CSF (503). ƒ_i_ = volume fraction of GW, WM, and CSF within the voxel. f_i_ = voxel total tissue fraction. α = theoretical uncorrected GABA concentration in GW (1), WM (0.5) and CSF (0). μ_i_ = group-average voxel fractions of GM, WM, and CSF.


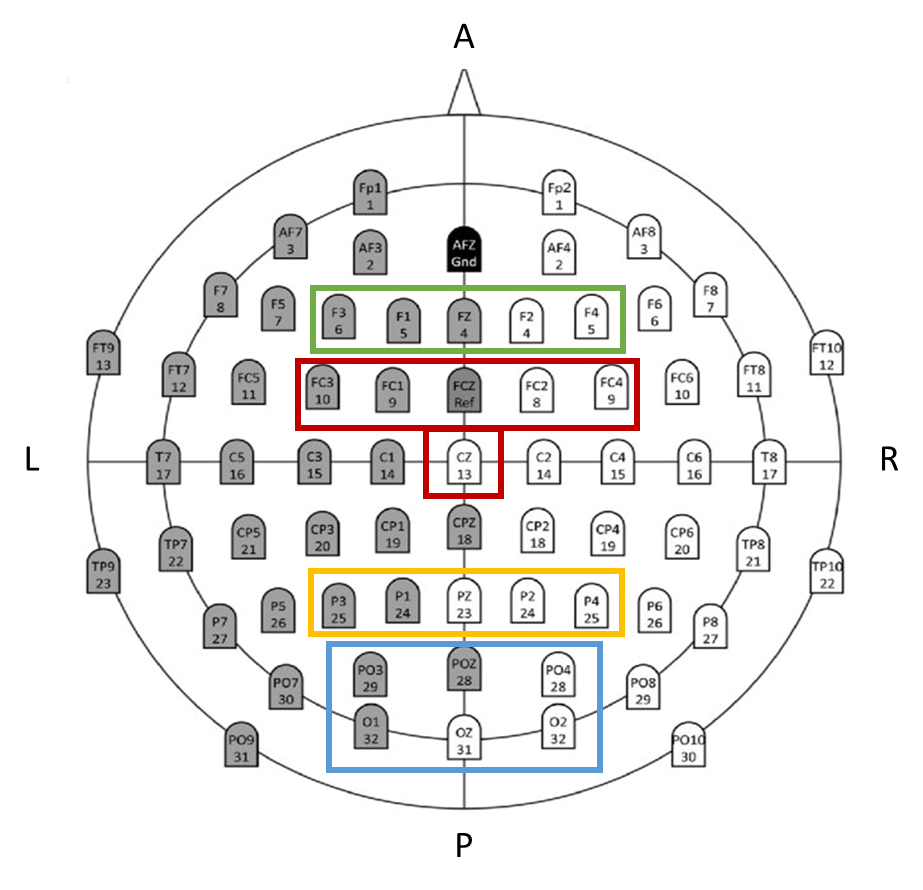


**Supplementary figure 2. Defined regions of interest for EEG data analyses.** Frontal region (Fz) in green, central region (Cz) in red, parietal region (Pz) in yellow and occipital region (Oz) in blue.


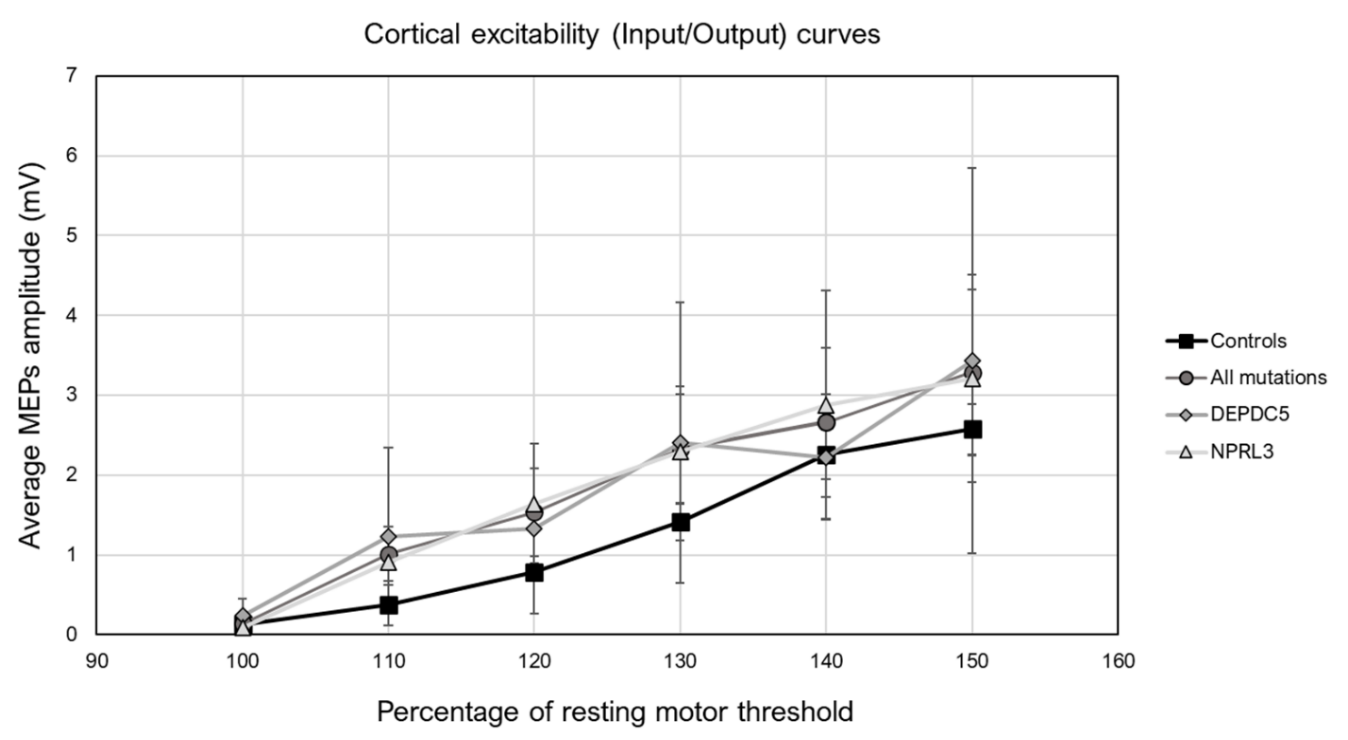


**Supplementary figure 3. Curves of cortical excitability.** For each percentage of resting motor threshold point, the average MEPs’ amplitude is represented as mean ± standard error.


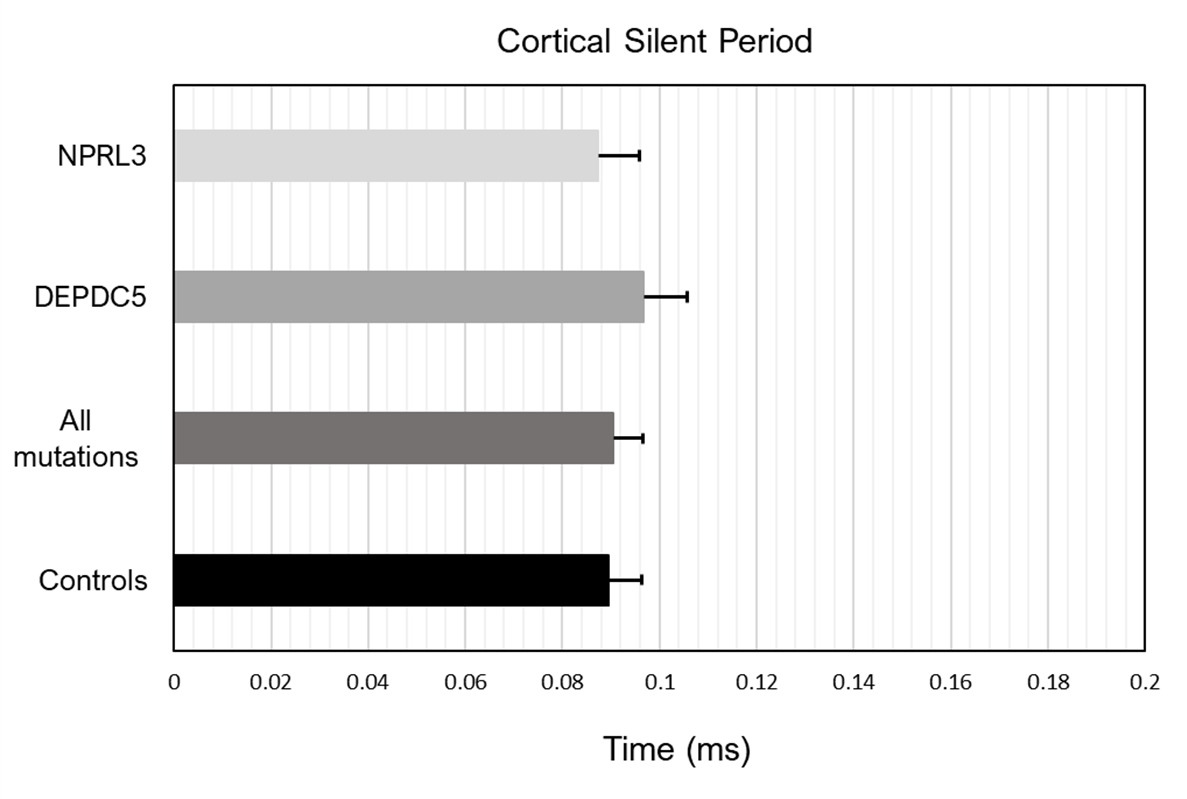


**Supplementary figure 4. Average cortical silent period in each group.** Values are indicated as mean ± standard error.


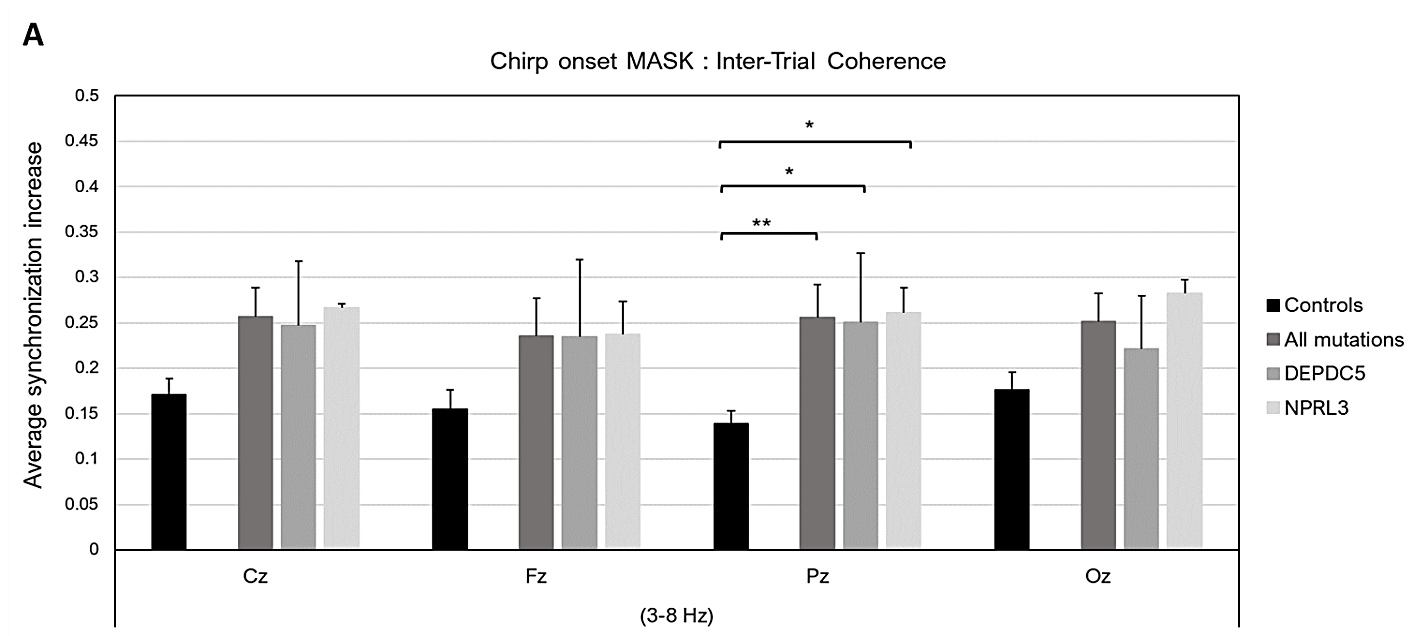


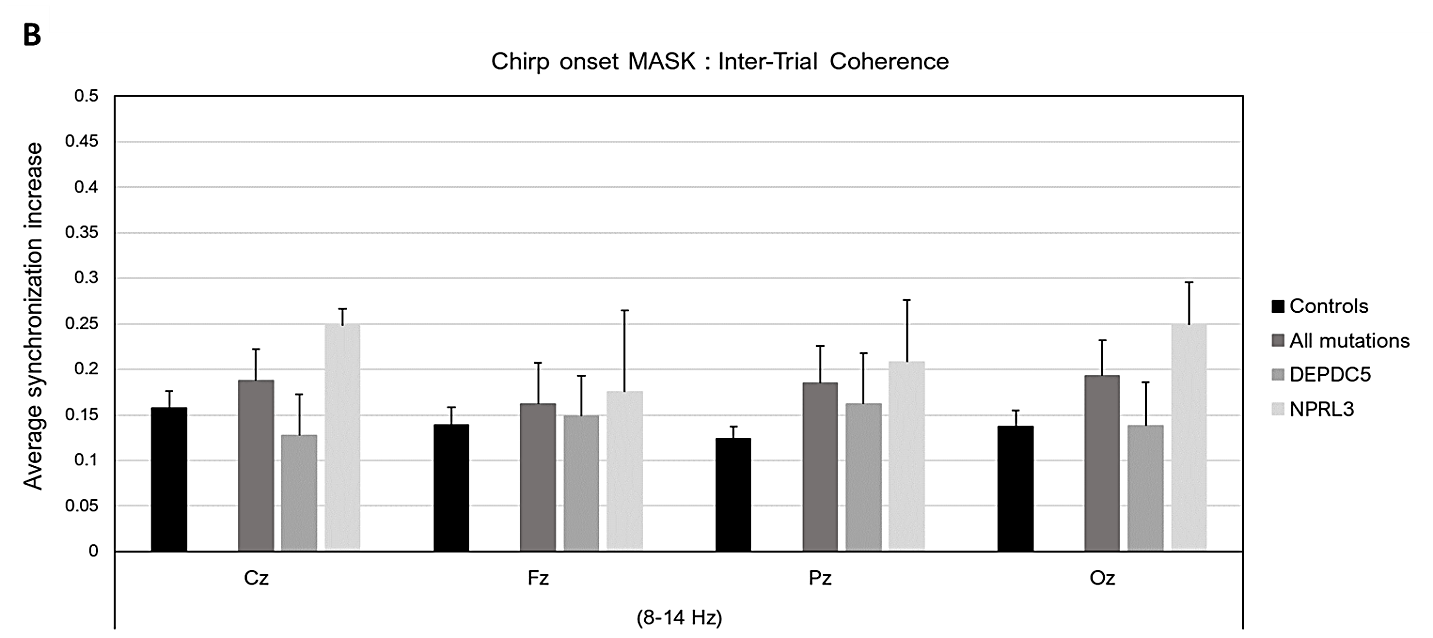


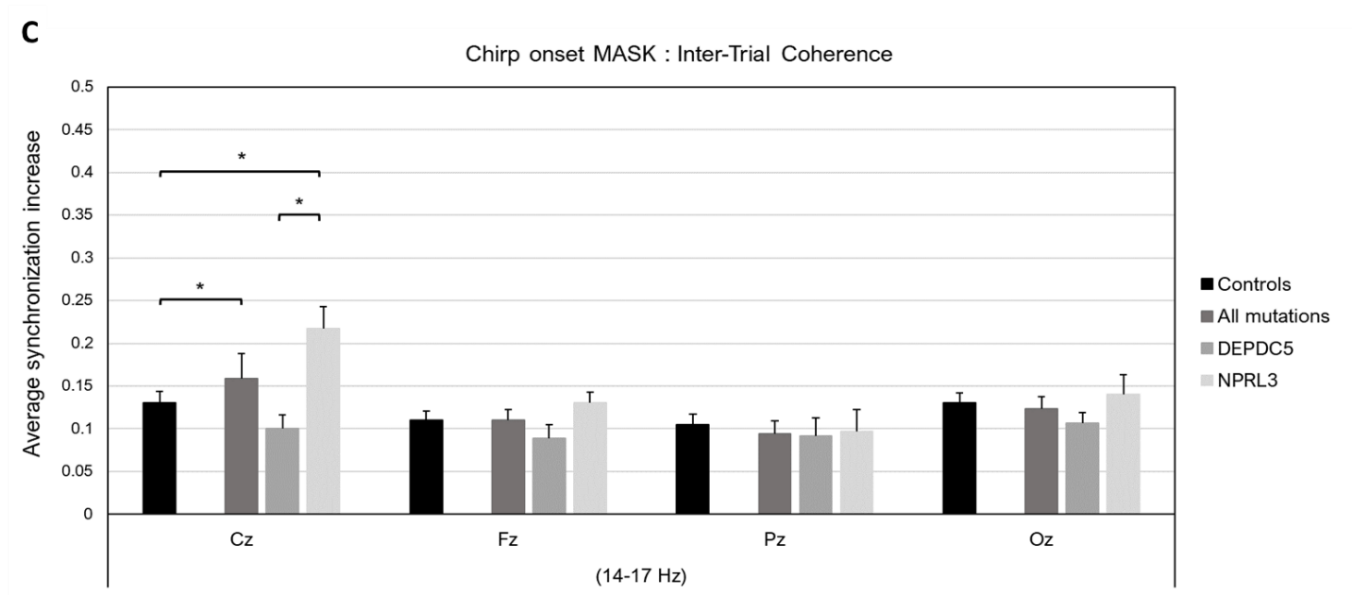


**Supplementary figure 5. Group-average changes in frequencies’ ITC during the Chirp onset.** Average synchronization increases are indicated as mean ± standard error.


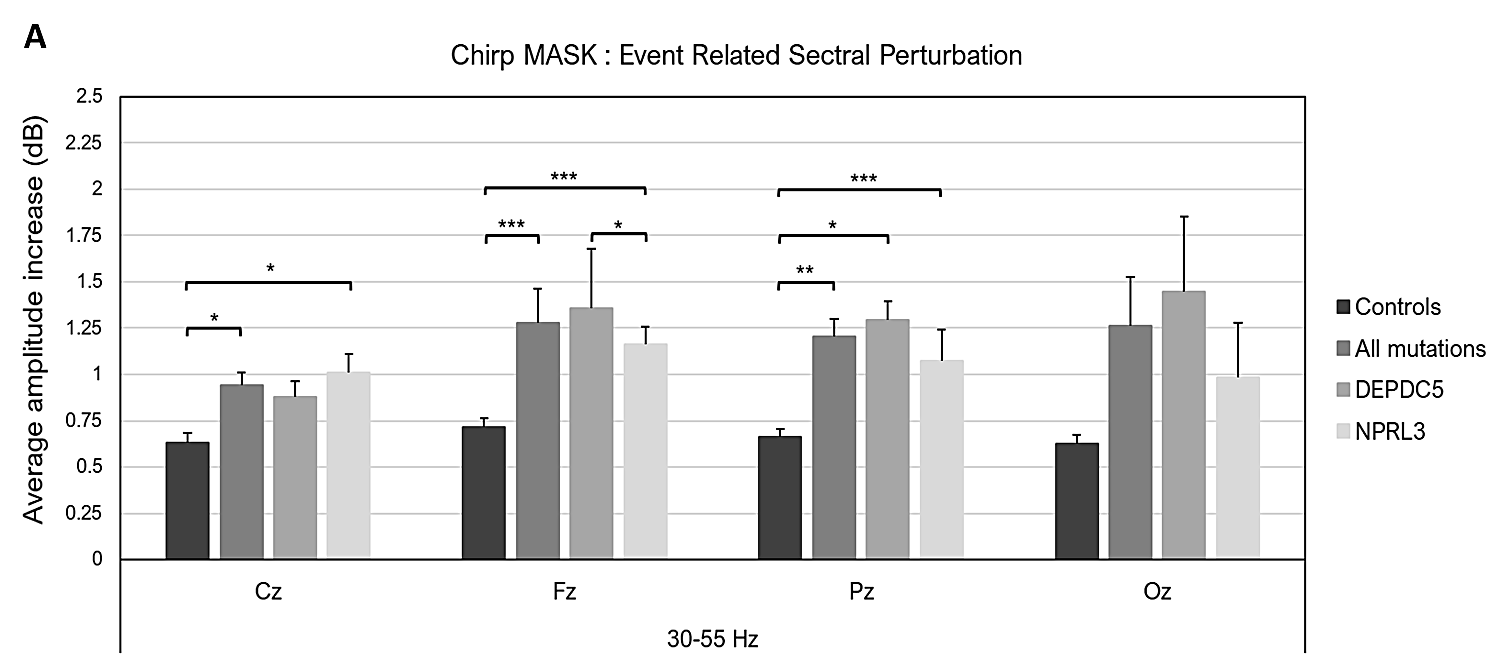


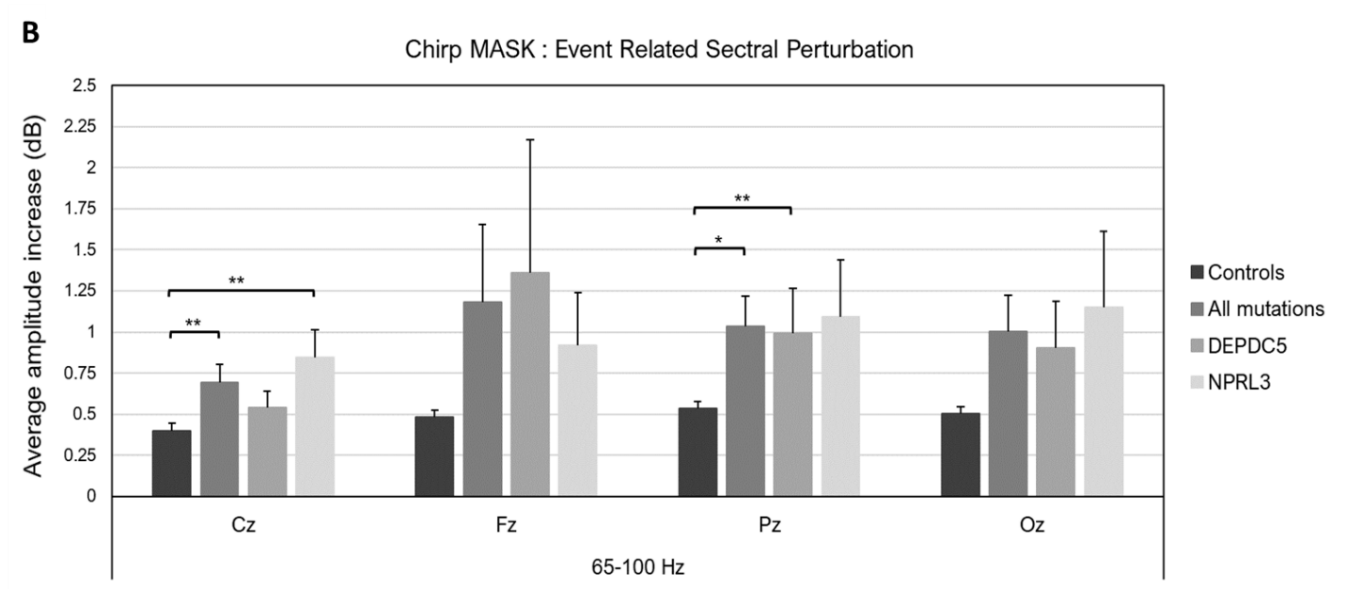


**Supplementary figure 6. Group-average changes in frequencies’ ERSP in response to the chirp sound.** Average amplitude increases are indicated as mean ± standard error.


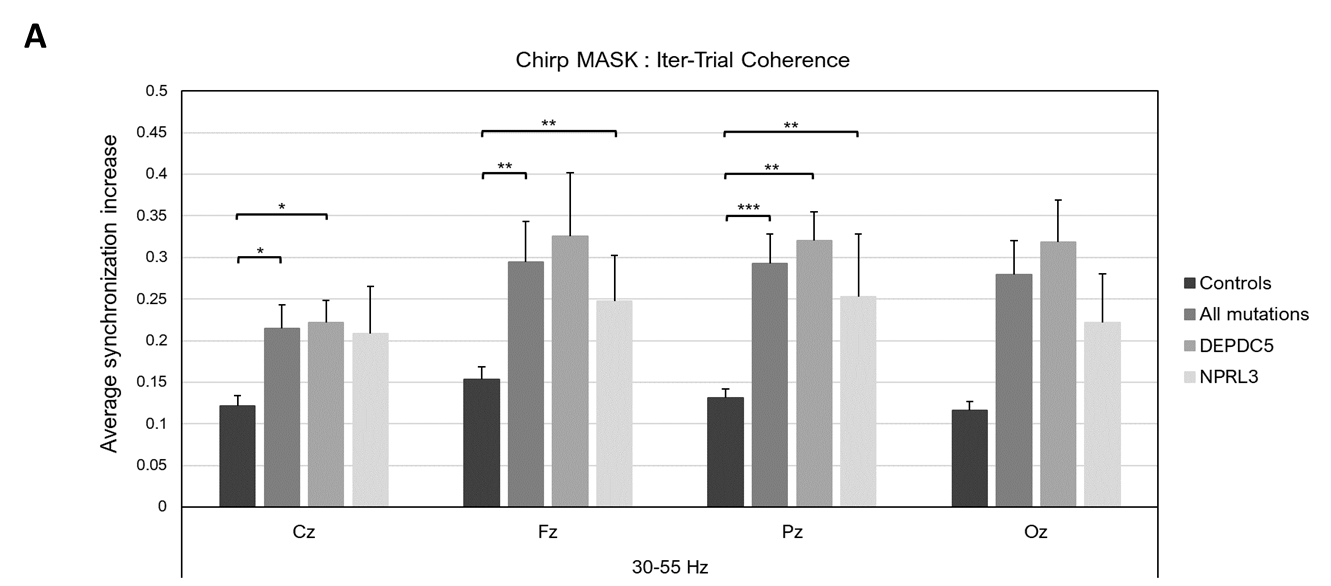


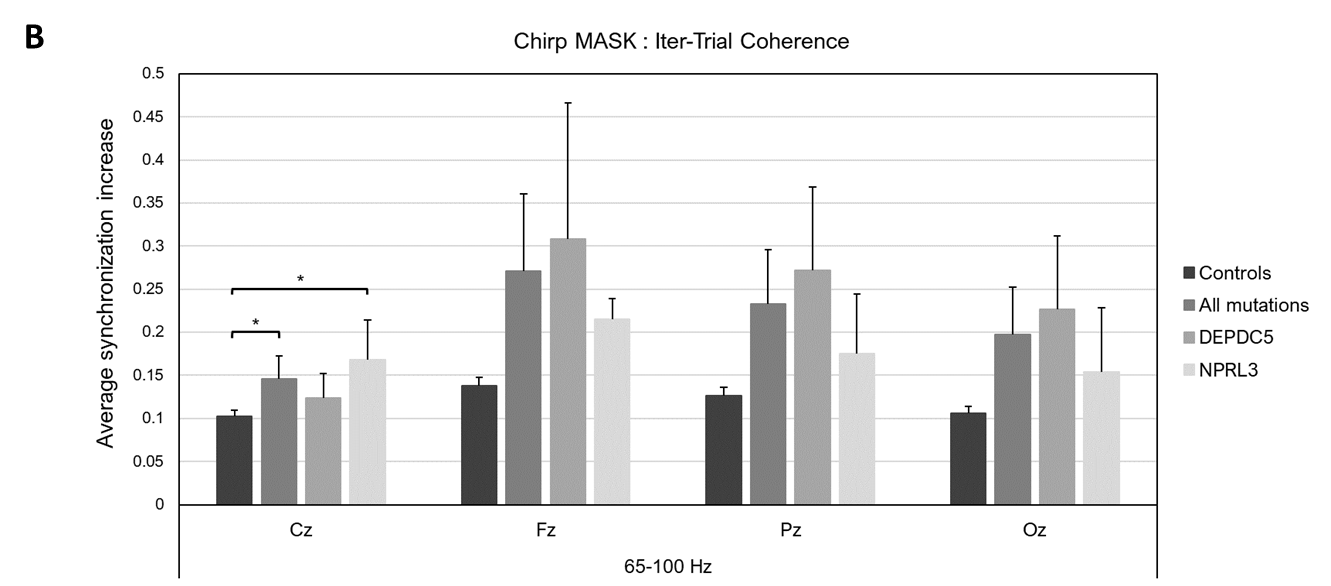


**Supplementary figure 7. Group-average changes in frequencies’ ITC in response to the chirp sound.** Average synchronization increases are indicated as mean ± standard error.
